# Factors affecting work-related non-fatal injuries among aged workers in South Korea

**DOI:** 10.3389/fpubh.2024.1260337

**Published:** 2024-01-22

**Authors:** Jungsun Park, Jong-shik Park, Younghoon Jung, Minoh Na, Yangho Kim

**Affiliations:** ^1^Department of Occupational Health, Catholic University of Daegu, Gyeongsan, Republic of Korea; ^2^Korea Labor Institute, Sejong-si, Republic of Korea; ^3^Department of Law, Pukyong National University, Busan, Republic of Korea; ^4^Korea Occupational Safety and Health Agency, Occupational Safety and Health Research Institute, Ulsan, Republic of Korea; ^5^Department of Occupational and Environmental Medicine, Ulsan University Hospital, University of Ulsan College of Medicine, Ulsan, Republic of Korea

**Keywords:** worker, older, work-related injury, Korea, non-fatal accidents, precarious employment

## Abstract

**Introduction:**

The objective of this paper is to investigate whether an aging workforce is associated with an increase in work-related non-fatal injuries and to explore the underlying reasons for this potential increase.

**Methods:**

Aged workers were defined as those who were at least 55-years-old. Work-related non-fatal injuries were assessed in aged and young workers who were registered with the workers’ compensation system from 2017 to 2021 of South Korea.

**Results:**

The mean estimated rate of work-related non-fatal injuries of aged workers (0.88/100) was about 2.5-times higher than that of younger workers (0.35/100). Most work-related non-fatal injuries in the older adults were in individuals working in the “construction sector” (36.0%), those with “elementary occupations (unskilled workers)” (45.0%), and those with employment status of “daily worker” (44.0%). “Trip & slip” (28.7%) and “falling” (19.6%) were more frequent types of work-related non-fatal injuries in aged workers relative to young workers. The category of “buildings, structures, and surfaces” was a more frequent cause of work-related non-fatal injuries in aged workers than young workers.

**Discussion:**

The incidence of non-fatal work-related injuries is higher among aged workers compared to their younger counterparts. The increased occurrence of aged workers participating in precarious employment and jobs, along with the greater physical vulnerability, is likely the cause of their higher rate of work-related non-fatal injuries.

## Introduction

The aging of South Korea’s population is occurring at a faster pace than any other country (1, 2). According to a demographic analysis, South Korea transitioned to an “aging society” in 2000, an “aged society” in 2018, and is projected to become a “super-aged” society by 2025 (1). This demographic shift is primarily attributed to the country’s low birth rate and the accelerated aging process. As a result, South Korea is anticipated to have the highest proportion of economically active individuals in 2025, followed by a subsequent decline. As a result, there is a growing expectation that the employment of older adults workers will rise to address the increasing labor shortages across various industries. Consequently, there is an ongoing upward trend in the percentage of workers aged 55 to 79 years (3). However, this aging workforce poses challenges in terms of workplace safety, as older adults employees are more susceptible to physiological declines in their sensory systems, equilibrium, and motor control (4–6). Therefore, there is a heightened need for policies that can effectively prevent occupational injuries among older workers in South Korea, given the impending labor shortages resulting from the rapid aging of the workforce.

Salminen (7) reviewed studies of non-fatal injuries and found that in 27% their results, there was no difference between older and younger workers; in 56%, younger workers are at most risk of a non-fatal injury; and the remaining 17%, older workers have more non-fatal injuries than younger workers. A comprehensive analysis of non-fatal injury data through a recent systematic review yielded the following results; out of the 57 studies reviewed, 49% (28 studies) indicated no significant correlation between workers’ age and non-fatal injuries. In 31% (18 studies), advancing age was even identified as a protective factor, suggesting that older workers experienced fewer non-fatal injuries compared to their younger counterparts. Conversely, 19% (11 studies) reported a higher risk of non-fatal injuries among older workers when compared to younger workers (8). Peng and Chan showed that the incidence of non-fatal accident among older workers is slightly (5.8%) lower than that of younger workers (9).

Earlier research conducted on the construction industry workforce indicated that advanced age at the time of injury was associated with increased injury-related costs, while the number of injuries was found to be unrelated to age. The elevated costs incurred by older workers can likely be attributed to the greater severity of their injuries (10–12). Therefore, younger workers may experience injuries more frequently, whereas older workers tend to sustain more severe injuries resulting in higher-cost claims (11).

These inconsistent findings may be attributed to the lack of a clear-cut definition for older workers and differences in the inclusion of industrial sectors as well as the analysis of injury severity among different studies (8).

The impact of workforce aging on work-related non-fatal injuries and the underlying reasons for the impact remain a subject of ongoing debate. The objective of this paper is to provide clarity on this matter.

## Methods

### Materials and subjects

An extensive PubMed search that covered publications on “older workers” and “work-related injury” since 2000 was conducted and the relevant papers and their references were evaluated initially. Among them, key references were finally cited.

“Aged worker” was at least 55-years-old, as defined by the Enforcement Decree of the Act on Prohibition of Age Discrimination in Employment and Aged Employment Promotion (13). Work-related non-fatal injuries were assessed in aged and young workers who were registered with the workers’ compensation system from 2017 to 2021 of South Korea.

Total waged workers, who were used as the denominator to estimate the work-related injury rates, were identified based on raw data of the Local Area Labor Force Survey from 2017 to 2021. The Local Area Labor Force Survey provides representative data on the age distribution, industry sector, occupational class, etc. of all waged workers. The number of participants in the survey ranged from 20,074,043 to 21,111,723.

### Variables

A total of 21 industrial sections in Korean Standard Industrial Classification (KSIC) (14), which is based on the International Standard Industrial Classification (ISIC) (15), were collapsed into 7 industrial sectors. The industrial sectors were “agriculture, forestry and fishing,” “mining and quarrying,” “manufacturing,” “construction,” “service,” “transportation and storage, information and communication,” and “others.” “Service” sector consists of “wholesale and retail trade”, “accommodation and food service activities”, “financial and insurance activities”, “real estate activities”, “professional, scientific, and technical activities”, “education”, “human health and social work activities”, and “arts, entertainment, and recreation”. Occupations were classified into 9 major groups in the Korean Standard Classification of Occupations (KSCO) (16) based on the International Standard Classification of Occupations ISCO (17). The occupational classes were “managers,” “professionals and related workers,” “clerks,” “service workers,” “sales workers,” “skilled workers related to agriculture, forestry and fisheries workers,” “craft and related trade workers,” “workers related to equipment, machine operating and assembling,” and “elementary occupations” (unskilled manual workers). Employment status was categorized as a contract for regular employment (≥1 year), temporary employment (1 month–<1 year), or daily employment (<1 month). Type of injuries in aged workers, listed in descending order of frequency, included “trip and slip,” “falling,” “getting caught,” “cutting, laceration, and puncture,” “collision,” “being struck by an object,” and “others.”

### Statistical analysis

The proportions of workplace non-fatal injuries in aged workers and younger workers were compared according to sex, industrial sector, occupational class, and employment status using a chi-squared test.

The number of aged workers who were registered for the workers’ compensation system is not directly available. Thus, the mean proportions of waged workers under 55-years-old (15,634,225) and 55-years-old or more (4,895,007) in Local Area Labor Force Survey from 2017 to 2021 (18) were applied to calculate the number of workers registered for workers’ compensation. The mean number of workers registered for workers’ compensation therefore consisted of 14,426,623 individuals who were under 55-years-old and 4,515,741 individuals who were 55-years-old or more. These numbers were used as the denominator to estimate the mean work-related injury rates of young and aged workers from 2017 to 2021. In the same manner, the number of young and aged workers registered for workers’ compensation in each industrial sector, occupational class, and employment status were also estimated. These numbers were used as the denominator to estimate the rates of non-fatal injuries according to industrial sector, occupation, and employment status. Relative risk with 95% confidence intervals (CIs) were obtained by dividing the rates of non-fatal injuries of aged workers by those of young workers to compare the rates of non-fatal injuries between young and aged workers.

SPSS version 20 (IBM Corp, Armonk, NY) was used for all statistical analyses, and a *p* value below 0.05 was considered significant.

## Results

We first compared the mean non-fatal work-related injuries among different age groups of men and women (Table 1). Aged workers accounted for 41.0% of work-related non-fatal injuries in men and 52.7% of work-related non-fatal injuries in women. Men comprised 80.4% of non-fatal injuries in non-aged workers, 71.8% of non-fatal injuries in aged workers, 76.6% of non-fatal injuries in overall workers. The average number of non-fatal injuries per year was 90,877.

**Table 1:** Mean non-fatal injuries according to age and gender.

Gender	Age < 55 yr	Age ≥ 55 yr	Proportion of aged workers	Relative risk (95% CI)
Men	41,127 (80.4%)	28,523 (71.8%)	41.0%	
	**0.51**	**1.17**		2.31 (2.27–2.34)
Women	10,051 (19.6%)	11,177 (28.2%)	52.7%	
	**0.16**	**0.54**		3.38 (3.29–3.47)
Total	51,177 (100.0%)	39,700 (100.0%)	43.7%	
	**0.35**	**0.88**		2.48 (2.45–2.51)

Non-bold numbers indicate *n* (%) of workers with non-fatal injuries, and bold numbers indicate *n*/100 workers. Workplace non-fatal injuries (%) in aged workers and younger workers were compared using the chi squared test for gender (*p* value < 0.001). The proportion of aged workers was calculated as the percentage (%) of aged workers among all workers with non-fatal injuries in each gender. Relative risk with 95% confidence intervals (CIs) were obtained by dividing the rate of non-fatal injuries of aged workers by those of young workers to compare the rates of non-fatal injuries between young and aged workers. CI, confidence interval.

The number of aged workers registered in the workers’ compensation system is not directly available. We therefore used the proportions of waged workers under 55-years-old and those 55-years-old or more of Local Area Labor Force Survey from 2017 to 2021 to estimate the number of workers who were registered for workers’ compensation. Based on these data, the mean estimated rate of work-related non-fatal injuries of aged workers (0.88/100) was about 2.5-times higher than that of younger workers (0.35/100) (Table 1). In addition, the estimated rate of work-related non-fatal injuries of all aged and young male workers was about two or three-times higher than those of all aged and young female workers.

We compared work-related non-fatal injuries in different industrial sectors (Table 2). Among aged workers, a largest proportion of work-related non-fatal injuries were in the “Construction” sector (36.0%), and this was followed by the “Service” sector (31.2%). However, the percentages of young workers with non-fatal injuries were greatest in the “Service” sector (34.7%) and then “Manufacturing” sector (29.6%). In addition, the proportion of aged workers in “Construction” sector was 57.1%. For the “Construction” sector, the estimated rate of work-related non-fatal injuries of both aged and younger workers was higher than average rate of those in overall industrial sectors (1.76/100 and 0.59/100 vs. 0.88/100 and 0.35/100). Moreover, the estimated rate of work-related non-fatal injuries of aged workers was approximately 3-times higher than that of younger workers (relative risk, 2.97) in the “Construction” sector. For the subsector of “Human health and social work activities”, which comprises 8.3 and 16.1% of the aged and younger workers in the “Service” sector, respectively, the estimated rate of work-related non-fatal injuries of both age groups was lower than the overall average rate in the “Service” sector (0.29/100 and 0.11/100 vs. 0.59/100 and 0.27/100). Although the estimated rate of work-related non-fatal injuries of aged and younger workers in “Mining and quarrying” sector was the highest, the proportion of aged workers in “Mining and quarrying” sector was the lowest among industrial sectors.

**Table 2:** Mean non-fatal injuries according to age and industrial sector.

Industrial sector	Age < 55 yr	Age ≥ 55 yr	Proportion of aged workers	Relative risk (95% CI)
Service	17,744 (34.7%)	12,380 (31.2%)	41.1%	
	**0.27**	**0.59**		2.19 (2.14–2.25)
Construction	10,745 (21.0%)	14,307 (36.0%)	57.1%	
	**0.59**	**1.76**		2.97 (2.90–3.05)
Mining and quarrying	50 (0.1%)	95 (0.2%)	65.4%	
	**0.79**	**2.08**		2.65 (1.90–3.75)
Manufacturing	15,130 (29.6%)	7,754 (19.5%)	33.9%	
	**0.45**	**1.12**		2.48 (2.42–2.55)
Agriculture, forestry, and fishing	537 (1.0%)	1,085 (2.7%)	66.9%	
	**0.69**	**1.06**		1.54 (1.39–1.70)
Transportation, storage, information, and communication	4,387 (8.6%)	1,488 (3.7%)	25.3%	
	**0.58**	**0.93**		1.59 (1.50–1.69)
Others	2,584 (5.0%)	2,591 (6.5%)	50.1%	
	**0.15**	**0.32**		2.09 (1.98–2.21)
Total	51,177 (100.0%)	39,700 (100.0%)	43.7%	
	**0.35**	**0.88**		2.48 (2.45–2.51)

Non-bold numbers indicate *n* (%) of workers with non-fatal injuries, and bold numbers indicate *n*/100 workers. Workplace non-fatal injuries (%) in aged workers and younger workers were compared using the chi squared test for industrial sector (*p* value < 0.001). The proportion of aged workers was calculated as the percentage (%) of aged workers among all workers with non-fatal injuries in each industrial sector. Relative risk with 95% confidence intervals (CIs) were obtained by dividing the rate of non-fatal injuries of aged workers by those of young workers to compare the rates of non-fatal injuries between young and aged workers. CI, confidence interval.

We also analyzed work-related non-fatal injuries according to occupational class (Table 3). Among aged workers, most work-related non-fatal injuries were for those with “elementary occupations” (45.0%), followed by “craft and related trade workers” (23.1%). Among young workers, these two classes were also responsible for most work-related non-fatal injuries. In addition, aged workers accounted for large percentages of workers employed in “elementary occupations” (46.5%), “skilled workers related to agriculture, forestry, and fisheries” (63.5%), and “craft and related trade workers” (49.7%). The highest estimated rate of work-related non-fatal injuries of aged workers was for those who were “skilled workers related to agriculture, forestry, and fisheries” (4.67/100), followed by “managers” (2.15/100) and “craft and related trade workers” (2.04/100). The estimated rates of work-related non-fatal injuries of young workers were also greatest for those employed as “skilled workers related to agriculture, forestry, and fisheries” (2.19/100), “managers” (1.84/100), and workers with “elementary occupations” (1.46/100). However, only a small proportion (less than 3%) of work-related non-fatal injuries were for “skilled workers related to agriculture, forestry, and fisheries” although the non-fatal injury rate in young and aged workers was highest among those workers. The estimated rates of work-related non-fatal injuries of aged workers employed in “elementary occupations” is similar to the average rate in overall occupational class.

**Table 3:** Mean non-fatal injuries according to age and occupation.

Occupation	Age < 55 yr	Age ≥ 55 yr	Proportion of aged workers	Relative risk (95% CI)
Managers	3,587 (7.3%)	2,842 (7.2%)	44.2%	
	**1.84**	**2.15**		1.17 (1.11–1.23)
Professionals and related workers	2,264 (4.6%)	1,025 (2.6%)	31.2%	
	**0.06**	**0.25**		4.15 (3.86–4.47)
Clerks	1,252 (2.5%)	203 (0.5%)	14.0%	
	**0.03**	**0.05**		1.41 (1.21–1.63)
Service workers	5,955 (12.1%)	3,877 (9.9%)	39.4%	
	**0.45**	**0.70**		1.56 (1.50–1.62)
Sales workers	963 (1.9%)	243 (0.6%)	20.1%	
	**0.07**	**0.10**		1.35 (1.17–1.55)
Skilled workers related to agriculture, forestry, and fisheries	648 (1.3%)	1,128 (2.9%)	63.5%	
	**2.19**	**4.67**		2.13 (1.94–2.35)
Craft and related trade workers	9,188 (18.6%)	9,067 (23.1%)	49.7%	
	**0.77**	**2.04**		2.66 (2.58–2.74)
Workers related to equipment, machine operating, and assembling	5,241 (10.6%)	3,252 (8.3%)	38.3%	
	**0.34**	**0.66**		1.95 (1.87–2.04)
Elementary occupations	20,322 (41.1%)	17,695 (45.0%)	46.5%	
	**1.46**	**0.99**		0.67 (0.66–0.69)
Total	49,420 (100.0%)	39,331 (100.0%)	44.3%	
	**0.34**	**0.87**		2.54 (2.51–2.58)

Non-bold numbers indicate *n* (%) of workers with non-fatal injuries, and bold numbers indicate *n*/100 workers. Workplace non-fatal injuries (%) in aged workers and younger workers were compared using the chi squared test for occupation (*p* value < 0.001). The proportion of aged workers was calculated as the percentage (%) of aged workers among all workers with non-fatal injuries in each occupation. Relative risk with 95% confidence intervals (CIs) were obtained by dividing the rate of non-fatal injuries of aged workers by those of young workers to compare the rates of non-fatal injuries between young and aged workers. CI, confidence interval.

We then compared work-related non-fatal injuries in different employment status (Table 4). The highest percentage of aged workers with work-related non-fatal injuries was for those employed as “regular workers” (51.5%) followed by “daily workers” (44.0%); the highest percentages for young workers were for those employed as “regular workers” (67.9%) followed by “daily workers” (27.4%). A comparison of all three categories of employment status showed that aged workers accounted for the largest percentage of “daily workers” (55.5%). Analysis of “daily workers” showed that the estimated rate of work-related non-fatal injuries in the aged (3.11/100) and younger workers (1.89/100) was much higher than the average rate in any other employment status or overall employment status.

**Table 4:** Mean non-fatal injuries according to age and employment status.

Employment status	Age < 55 yr	Age ≥ 55 yr	Proportion of aged workers	Relative risk (95% CI)
Regular	34,472 (67.9%)	20,352 (51.5%)	37.1%	
	**0.32**	**0.89**		2.82 (2.77–2.87)
Temporary	2,386 (4.7%)	1,761 (4.5%)	42.5%	
	**0.08**	**0.10**		1.24 (1.16–1.31)
Daily	13,928 (27.4%)	17,401 (44.0%)	55.5%	
	**1.89**	**3.11**		1.65 (1.61–1.66)
Total	50,786 (100.0%)	39,514 (100.0%)	43.8%	
	**0.35**	**0.88**		2.49 (2.45–2.52)

Non-bold numbers indicate *n* (%) of workers with non-fatal injuries, and bold numbers indicate *n*/100 workers. Workplace non-fatal injuries (%) in aged workers and younger workers were compared using the chi squared test for employment status (*p* value < 0.001). The proportion of aged workers was calculated as the percentage (%) of aged workers among all workers with non-fatal injuries in each employment status. Relative risk with 95% confidence intervals (CIs) were obtained by dividing the rate of non-fatal injuries of aged workers by those of young workers to compare the rates of non-fatal injuries between young and aged workers. CI, confidence interval.

Analysis of the types of accidents responsible for work-related non-fatal injuries (Table 5) showed that “trip & slip” (28.7%) was the most common cause, followed by “falling” (19.6%) in aged workers, however, “trip & slip” (16.8%) and “getting caught” (16.6%) were the major causes in young workers. According to further analysis by industrial sectors, “trip & slip” (48.0%) and “falling” (33.7%) were the most common cause in aged workers of “service” and “construction” sector, respectively, whereas “getting caught” (16.6%) was the major causes in young workers of “manufacturing” sector (data not shown). Aged workers accounted for large percentages of “trip & slip” (57.0%) and “falling” (53.8%) as the types of accidents.

**Table 5:** Accident types responsible for mean non-fatal injuries.

	Age < 55 yr	Age ≥ 55 yr	Proportion of aged workers
Trip, slip	8,594 (16.8%)	11,413 (28.7%)	57.0%
Falling	6,653 (13.0%)	7,762 (19.6%)	53.8%
Getting caught	8,473 (16.6%)	4,496 (11.3%)	34.7%
Cutting, laceration, puncture	6,417 (12.5%)	3,749 (9.4%)	36.9%
Collision	4,282 (8.4%)	3,144 (7.9%)	42.3%
Being struck by an object	3,984 (7.8%)	3,325 (8.4%)	45.5%
Others	12,774 (25.0%)	5,811 (14.6%)	31.3%
Total	51,177 (100.0%)	39,700 (100.0%)	43.7%

Workplace non-fatal injuries (%) in aged workers and younger workers were compared using the chi squared test for accident type (*p* value < 0.001). The proportion of aged workers was calculated as the percentage (%) of aged workers among all workers with non-fatal injuries in each accident type.

We also analyzed the specific agents responsible for non-fatal injuries (Table 6). The main agent in aged and young workers was “buildings, structures, and surfaces (e.g., floor, steps, various building, and structural components, etc.)” (45.2 and 30.4%, respectively). However, “equipment and machinery” accounted for larger percentage in young workers (21.5%) than in aged workers (15.8%). Aged workers accounted for large percentages of “buildings, structures, and surfaces” (53.6%) as specific agents for injuries, whereas younger workers accounted for larger percentages of “equipment and machinery” (63.6%).

**Table 6:** Agents responsible for mean non-fatal injuries.

Fatal injury-causing agent	Age < 55 yr	Age ≥ 55 yr	Proportion of aged workers
Buildings, structures, and surfaces (e.g., floor, steps, various building and structural components, etc.)	15,544 (30.4%)	17,944 (45.2%)	53.6%
Equipment and machinery	11,010 (21.5%)	6,291 (15.8%)	36.4%
Parts, accessories, and materials	6,646 (13.0%)	4,980 (12.5%)	42.8%
Means of transportation	5,647 (11.0%)	2,637 (6.6%)	31.8
Others	12,330 (24.1%)	7,848 (19.8%)	38.9%
Total	51,177 (100.0%)	39,700 (100.0%)	43.7%

Workplace non-fatal injuries (%) in aged workers and younger workers were compared using the chi squared test for causing agent (*p* value < 0.001). The proportion of aged workers was calculated as the percentage (%) of aged workers among all workers with non-fatal injuries in each causing agent.

## Discussion

Previous studies have shown inconsistent findings regarding the association between workforce aging and work-related injuries (8). In our present study, we observed a significantly higher mean estimated rate of work-related non-fatal injuries per 100 workers among aged workers (0.88) compared to young workers (0.35). Our study utilized a representative national workers’ compensation database for South Korea, encompassing all industrial sectors and occupations over the past 5 years. These findings indicate that aged workers have a higher incidence of work-related non-fatal injuries compared to their younger counterparts. Two factors likely contribute to this increased incidence of injuries among aged workers in comparison to their younger counterparts.

First, the increased occurrence of aged workers participating in precarious employment and jobs likely contribute to their higher rate of non-fatal injuries. Our study of aged workers in South Korea from 2017 to 2021 revealed that the highest percentages of work-related non-fatal injuries were observed among workers in the “construction sector” (36.0%) and those in “elementary occupations” (unskilled workers) (45.0%). Additionally, a significant proportion of aged workers were employed in the “construction sector” (57.1%) and held an employment status of “daily worker” (55.5%). These findings indicate that work-related non-fatal injuries among aged workers are more likely to occur in industrial sectors, occupations, and employment statuses with a higher concentration of aged workers. In the present study, “construction sector” and employment status of “daily worker” were identified as precarious, as they exhibited higher rates of non-fatal injuries among young workers as well as aged workers. Consequently, aged workers who have precarious employment and jobs that expose them to greater occupational safety and health risks are more susceptible to work-related non-fatal injuries. Our previous publication also found that “elementary occupations” had the largest proportion of aged workers (19). Bravo et al. similarly found that the higher injury rate among aged workers may be attributed to the type of industry and employment contract, and nature of the occupation (8). For instance, industries such as construction and agriculture, which employ a substantial number of aged workers, typically experience higher fatality rates (8, 20). This suggests that employment in precarious employment and jobs makes aged workers more vulnerable to workplace injuries.

Second, the greater physical vulnerability of aged workers is likely the cause of their higher injury rate. Trip/slip and fall are the most common cause of non-fatal injuries (21, 22). Kemmlert and Lundholm conducted an analysis of the Swedish Occupational Injury Information System, which encompassed various industrial sectors. Their findings revealed that male workers aged 45 years and above had a higher occurrence of slip, trip, and fall incidents compared to workers under the age of 45 (23). In another investigation by Coantonio et al., workers’ compensation data from Ontario, Canada, was examined. The study revealed that falls accounted for 76% of traumatic brain injury (TBI) claims among construction workers aged 55 to 64, whereas only 45% of claims from workers aged 17 to 24 were related to TBI (23, 24). Furthermore, Schoenfisch et al. (25) found that injuries among more senior workers were more likely to cause serious problems that required longer hospitalization stays, indicating a slower recovery from injury. The present national representative study found that “trip/slip” and “falling” were the most common type of work-related non-fatal injury in aged workers than young workers. “Trip/slip” and “falling” may be related to the more limited physical abilities (muscle strength and agility) of aged workers. The physical, mental and motor skills (movement of arms, hands and legs) changes related to aging may affect older workers’ performance and their health and safety. Age-related decline in lower extremity muscle strength increases likelihood of slips and falls. Age-related decline in balance control, for which sensory inputs such as vision, proprioception, and vestibular sensations are important, are also associated with a greater risk of slips or falls (4, 6, 26). Furthermore, age-related osteoporotic bones are more likely to break as a result of workplace slips or falls. Our analysis of injury-causing agents also showed that “buildings, structures, and surfaces” was a more common cause of work-related non-fatal injuries in aged workers than young workers. In a study using self-reported data from injured carpentry workers (*n* = 4,429), the contributing factors to falls from the same level (“Trip/slip”) were found to be tripping over debris, difficult work terrain (rocky, muddy, uneven), the slope of the lot, lack of backfill around the foundation, and difficult access and/or egress from the building (27). Taken together, these results strongly suggest that the physical limitations of older adults workers were responsible for their higher frequency of occupational injuries. Other studies also reported that aged workers who had poor muscle strength and elasticity and limited range of joint motion were more likely to suffer from work-related fatal injuries (4, 28, 29).

Taken together, the increased occurrence of aged workers participating in precarious employment and jobs, along with the greater physical vulnerability, is likely the cause of their higher rate of work-related non-fatal injuries. This finding aligns with prior research, which similarly identified that precarious employment and jobs render aged workers more vulnerable to work-related injuries due to age-related declines in physical capabilities (8, 20).

Thus, implementing exercise programs aimed at improving the physical functional capacities, particularly balance and muscle strength, among aged workers, could prove beneficial in reducing the frequency of workplace injuries. For example, older workers should start low extremity muscle strengthening such as seated calf raises, seated knee lifts and leg lifts, seated leg extensions, standing leg lifts and sit-to-stand exercise, and then progress to squatting and core strengthening tailored to muscle strength status. They should also perform series of balance exercises such as standing with feet apart/together with eyes open, and then standing on one foot, with eyes open/closed based on balance control status.

The age structure of the workforce is currently changing significantly, defining the needs for creating age-friendly working conditions is becoming a priority in many countries. We can identify good practices in several countries. In USA, the National Institute of Occupational Safety and Health (NIOSH) National Center for Productive Aging and Work seeks to advance lifelong well-being for workers of all ages and supports productive aging across the working life (30). The Center continues to work on such important issues as how organizations are addressing the needs of an aging workforce and identifying interventions and strategies to support both workers of all age groups and organizations that employ them. The European Agency for Safety and Health at Work coordinated the European campaign “Healthy workplaces for all ages” to promote sustainable work and healthy aging from the start of a person’s working life, and to prevent health problems and enable people to work for longer in 2016–2017 (31). Japanese government has developed and disseminated aging-friendly occupational health and safety guidelines with action checklist and good practices to prevent work-related injury in older workers according to industrial sectors (32).

The present study has several strengths. Firstly, it was based on a representative national workers’ compensation database for South Korea that encompasses all industrial sectors and occupations. Secondly, it estimated the incidences of work-related non-fatal injuries among aged workers in comparison to their younger counterparts.

Our research also had several limitations. Firstly, due to the cross-sectional design of the study, we are unable to establish causal relationships. Secondly, the precise incidence rates of work-related non-fatal injuries among aged workers could not be calculated due to the unavailability of the exact age distribution for all workers registered under the workers’ compensation scheme. Consequently, our data provides estimates of the incidence of work-related non-fatal injuries based on information derived from a representative Local Area Labor Force Survey. Finally, aging is not easily distinguishable between age groups. There is no universally agreed-upon age at which someone becomes an “aged worker.” Instead, it is often defined based on the specific needs, policies, or research objectives of a particular group or institution. Consequently, our results address the characteristics of non-fatal injuries among workers aged 55 years or older compared to those under 55 years, rather than among “aged workers” in a general sense.

The present study holds significant practical implications for mitigating the occurrence of work-related injuries. Firstly, it emphasizes the importance of directing greater attention towards aged workers, given their higher susceptibility to such injuries compared to their younger counterparts. Secondly, it underscores the necessity for improving occupational safety and health measures specifically tailored to individuals engaged in precarious employment and jobs, as a considerable number of aged workers fall into this category. Thirdly, it highlights the need for industries and occupations with a substantial aging workforce to establish a work environment that addresses the increased physical vulnerabilities of aged workers.

In conclusion, the number of aged workers in South Korea engaging in various economic activities and working as waged employees is consistently rising. The incidence of non-fatal work-related injuries is higher among aged workers compared to their younger counterparts. Combined with engagement in precarious employment and jobs, frailty likely contribute to having higher rate of work-related non-fatal injuries in aged workers. To mitigate workplace injuries, it is crucial for employers to enhance safety and health standards specifically tailored to the employment types and jobs most common among aged workers. Additionally, implementing preventive measures that enhance the physical and functional capabilities of aged workers, particularly focusing on balance and muscle strength, can play a vital role in averting work-related injuries in this demographic.

## Data availability statement

The data analyzed in this study is subject to the following licenses/restrictions: Korea Occupational Safety and Health Agency. Requests to access these datasets should be directed to MN, mino05@kosha.or.kr.

## Ethics statement

The studies involving humans were approved by Institutional Review Board of Ulsan University Hospital. The studies were conducted in accordance with the local legislation and institutional requirements. The participants provided their written informed consent to participate in this study.

## Author contributions

JP: Writing – original draft. J-sP: Methodology, Writing – review & editing. YJ: Investigation, Writing – review & editing. MN: Investigation, Writing – review & editing. YK: Conceptualization, Writing – review & editing.

## References

[1] Statistics Korea. Population projections (2022). Available from: http://kostat.go.kr/portal/korea/kor_nw/2/1/index.board?bmode=read&aSeq=252623.

[2] United Nations. World population prospects 2022. (2022). Available at: https://population.un.org/wpp/.

[3] Korea Employment Information Service. Medium and long-term labor force and employment projections 2018–2028 handbook. Chungcheongbuk-Do, Korea: Korea Employment Information Service (2020).

[4] Lockhart TE Smith JL Woldstad JC. Effects of aging on the biomechanics of slips and falls. Hum Factors. (2005) 47:708–29. doi: 10.1518/001872005775571014, PMID: 16553061 PMC2895260

[5] Rhee K Seo D. Gender-specific incidence rate of industrial accidents and type of accident. OSH Issue Rep. (2014) 8:2–29.

[6] Punakallio A. Balance Abilities of Different-Aged Workers in Physically Demanding Jobs. J Occup Rehabil. (2003) 13:33–43. doi: 10.1023/a:1021845823521, PMID: 12611029

[7] Salminen S. Have young workers more injuries than older ones? An international literature review. J Saf Res. (2004) 35:513–21. doi: 10.1016/j.jsr.2004.08.005, PMID: 15530925

[8] Bravo G Viviani C Lavallière M Arezes P Martínez M Dianat I. Do older workers suffer more workplace injuries? A systematic review. Int J Occup Saf Ergon. (2022) 28:398–427. doi: 10.1080/10803548.2020.1763609, PMID: 32496932

[9] Peng L Chan A. A Meta-analysis of the relationship between ageing and occupational safety and health. Saf Sci. (2019) 112:162–72. doi: 10.1016/j.ssci.2018.10.030

[10] Schwatka NV Butler LM Rosecrance JR. An aging workforce and its effect on injury in the construction industry. Epidemiol Rev. (2012) 34:156–67. doi: 10.1093/epirev/mxr020, PMID: 22173940 PMC7735369

[11] Mallon TM Cherry SE. Investigating the relationship between worker demographics and nature of injury on Federal Department of Defense Workers' compensation injury rates and costs from 2000 to 2008. J Occup Environ Med. (2015) 57:S27–30. doi: 10.1097/jom.0000000000000416, PMID: 25741611

[12] Shuford H Restrepo T. Thinking about an aging workforce—Potential impact on workers compensation. Florida, USA: National Council on Compensation Insurance (NCCI) (2005). 1 p.

[13] Enforcement decree of the act on prohibition of age discrimination in employment and aged employment promotion. Korea Legistration Research Institute. (2020). Available at: https://elaw.klri.re.kr/kor_service/lawView.do?hseq=60976&lang=ENG.

[14] Korean Standard Industrial Classification (KSIC). Statistics Korea (2017) Available at: http://kssc.kostat.go.kr/ (Accessed April 1, 2019).

[15] The International Standard Industrial Classification (ISIC). United Nations statistics division (2008). Available at: https://unstats.un.org/unsd/classifications/Econ/isic (Accessed April 1, 2019).

[16] Korean Standard Classification of Occupations (KSCO). Statistics Korea (2016). Available at: http://kssc.kostat.go.kr/ (Accessed April 1, 2019).

[17] International Labour Office. International standard classification of occupations: ISCO-88. Geneva: ILO (2012). Available at: http://www.ilo.org/public/english/bureau/stat/isco/isco08/index.htm.

[18] Statistics Korea. Local Area Labor Force Survey. (2023). Available at: http://kosis.kr/statisticsList/statisticsList_01List.jsp?vwcd=MT_ZTITLE&parentId=B#SubCont.

[19] Park J Kim SG Park JS Han B Kim KB Kim Y. Hazards and health problems in occupations dominated by aged workers in South Korea. Ann Occup Environ Med. (2017) 29:27. doi: 10.1186/s40557-017-0177-9, PMID: 28670457 PMC5485698

[20] Smith SM Pegula SM. Fatal occupational injuries to older workers. Mon Labor Rev. January 2020 (2020):1–13. doi: 10.21916/mlr.2020.2

[21] Dement JM Lipscomb H. Workers' compensation experience of North Carolina residential construction workers, 1986–1994. Appl Occup Environ Hyg. (1999) 14:97–106. doi: 10.1080/104732299303269, PMID: 10457636

[22] Friedman LS Forst LS. Workers' compensation costs among construction workers: a robust regression analysis. J Occup Environ Med. (2009) 51:1306–13. doi: 10.1097/JOM.0b013e3181ba46bb, PMID: 19749601

[23] Kemmlert K Lundholm L. Slips, trips and falls in different work groups--with reference to age and from a preventive perspective. Appl Ergon. (2001) 32:149–53. doi: 10.1016/s0003-6870(00)00051-x 11277507

[24] Colantonio A McVittie D Lewko J Yin J. Traumatic brain injuries in the construction industry. Brain Inj. (2009) 23:873–8. doi: 10.1080/02699050903036033, PMID: 20100123

[25] Schoenfisch AL Lipscomb HJ Shishlov K Myers DJ. Nonfatal construction industry-related injuries treated in hospital emergency departments in the United States, 1998–2005. Am J Ind Med. (2010) 53:570–80. doi: 10.1002/ajim.20829, PMID: 20506460

[26] Government of Alberta Labour. A guide to managing an aging workforce. Alberta, Canada: Government of Alberta Labour (2006).

[27] Lipscomb HJ Dement JM Li L Nolan J Patterson D. Work-related injuries in residential and drywall carpentry. Appl Occup Environ Hyg. (2003) 18:479–88. doi: 10.1080/10473220301422, PMID: 12746070

[28] de Zwart BC Frings-Dresen MH van Dijk FJ. Physical workload and the aging worker: a review of the literature. Int Arch Occup Environ Health. (1995) 68:1–12. doi: 10.1007/bf01831627, PMID: 8847107

[29] Maertens JA Putter SE Chen PY Diehl M Huang Y-H. Physical capabilities and occupational health of older workers In: Borman WC Hedge JW, editors. The Oxford handbook of work and aging. Oxford, England: Oxford University Press (2012)

[30] Productive Aging and Work. National Institute for Occupational Safety and Health (2023). Available at: https://www.cdc.gov/niosh/topics/productiveaging/ncpaw.html.

[31] Healthy Workplaces for All Ages. The European Agency for Safety and Health at work (2016). Available at: https://healthy-workplaces.osha.europa.eu › campaign.

[32] Ageing-Friendly Occupational Health and Safety Guidelines with Action Checklist and Good Practices. Japanese Ministry of Health, Labour and Welfare (2017). Available at: https://www.mhlw.go.jp › gyousei › anzen.

